# Participation of 3‐*O*‐sulfated heparan sulfates in the protection of macrophages by herpes simplex virus‐1 glycoprotein D and cyclophilin B against apoptosis

**DOI:** 10.1002/2211-5463.12145

**Published:** 2016-12-24

**Authors:** Maxime Delos, Charles Hellec, François Foulquier, Mathieu Carpentier, Fabrice Allain, Agnès Denys

**Affiliations:** ^1^ Unité de Glycobiologie Structurale et Fonctionnelle (UGSF)UMR 8576CNRSUniversity of LilleFrance

**Keywords:** apoptosis, cyclophilin, heparan sulfates, HSV‐1 glycoprotein D, macrophages

## Abstract

Heparan sulfates (HS) are involved in numerous biological processes, which rely on their ability to interact with a large panel of proteins. Although the reaction of 3‐O‐sulfation can be catalysed by the largest family of HS sulfotransferases, very few mechanisms have been associated with this modification and to date, only glycoprotein D (gD) of herpes simplex virus‐1 (HSV‐1 gD) and cyclophilin B (CyPB) have been well‐described as ligands for 3‐*O*‐sulfated HS. Here, we hypothesized that both ligands could induce the same responses via a mechanism dependent on 3‐*O*‐sulfated HS. First, we checked that HSV‐1 gD was as efficient as CyPB to induce the activation of the same signalling events in primary macrophages. We then demonstrated that both ligands efficiently reduced staurosporin‐induced apoptosis and modulated the expression of apoptotic genes. In addition to 3‐*O*‐sulfated HS, HSV‐1 gD was reported to interact with other receptors, including herpes virus entry mediator (HVEM), nectin‐1 and ‐2. Thus, we decided to identify the contribution of each binding site in the responses triggered by HSV‐1 gD and CyPB. We found that knock‐down of 3‐*O*‐sulfotransferase 2, which is the main 3‐*O*‐sulfated HS‐generating enzyme in macrophages, strongly reduced the responses induced by both ligands. Moreover, silencing the expression of HVEM rendered macrophages unresponsive to either HSV‐1 gD and CyPB, thus indicating that both proteins induced the same responses by interacting with a complex formed by 3‐*O*‐sulfated HS and HVEM. Collectively, our results suggest that HSV‐1 might hijack the binding sites for CyPB in order to protect macrophages against apoptosis for efficient infection.

**Abbreviations**
2‐OST: 2‐*O*‐sulfotransferase
3‐OST: 3‐*O*‐sulfotransferase
6‐OST: 6‐*O*‐sulfotransferase
CyPB: cyclophilin B
ERK: extracellular signal‐regulated kinases
gD: glycoprotein D
GlcNAc: *N*‐acetylglucosamine
GlcUA: glucuronic acid
HPRT: hypoxanthine‐guanine phosphoribosyl transferase
HS: heparan sulfates
HSV‐1: herpes simplex virus‐1
HVEM: herpes virus entry mediator
IdoUA: l‐iduronic acid
NDST: *N*‐deacetylase/*N*‐sulfotransferase
NF‐κB: nuclear factor‐kappa B
PI: propidium iodide
RT‐PCR: reverse transcription‐polymerase chain reaction
siRNA: small‐interfering RNA
TBS: Tris buffer saline

Heparan sulfates (HS) are sulfated polysaccharides composed of alternating glucosamine (GlcN) and uronic acid (GlcUA/IdoUA) residues. These repeating disaccharide units are clustered in a series of domains of relatively high IdoUA content and sulfate density (NS domains), bound by short transition zones with intermediate sulfation patterns and separated by N‐acetylated domains (NA domains). HS are involved in a plethora of biological processes, which rely on their ability to selectively interact with a large panel of proteins 1, 2. HS–protein interactions are mainly dependent on the density and position of sulfate groups into the HS structure, which result in sequential actions of many HS biosynthetic enzymes. HS are initially synthesized as a linear polymer composed of alternating GlcUA and *N*‐acetylglucosamine (GlcNAc) units, which is then subjected to enzymatic modifications in the Golgi apparatus. In the stepwise scheme of HS biosynthesis, the precursor is first subject to the action of *N*‐deacetylases/*N*‐sulfotransferases (NDSTs), which convert GlcNAc to N‐sulfated GlcN (GlcNS) residues. This crucial reaction creates the prerequisite substrates needed for the next enzymatic modifications. The further modifications include C5‐epimerization of some GlcUA into IdoUA residues and 2‐O‐sulfation of uronic acid residues. These steps are catalysed, respectively, by C5‐epimerase and 2‐*O*‐sulfotransferase (2‐OST). The latter introduces a sulfate group in position 2 of mainly IdoUA and rarely GlcUA. Finally, the reactions catalysed by 6‐*O*‐sulfotransferases (6‐OSTs) and 3‐*O*‐sulfotransferases (3‐OSTs) consist in the addition of sulfate groups to the 6‐OH and 3‐OH positions of GlcN residues respectively 1, 3, 4. Importantly, NDSTs, 6‐OSTs and 3‐OSTs are represented by distinct isoenzymes, which exhibit fine differences in substrate specificity and for which the expression is dependent on cell type and tissue environment. For example, the 3‐OST family is represented by seven isoenzymes in human (3‐OST1, 2, 3A, 3B, 4, 5 and 6), which possess more than 60% of sequence homology in the sulfotransferase domain. While 3‐OST1 was reported to generate an HS‐binding site for antithrombin‐III, 3‐OST2, 3A, 3B, 4 and 6 transfer sulfate groups to the 3‐OH position of GlcNS or GlcNH_2_ adjacent to an IdoUA2S residue, thus providing HS‐binding sites for the glycoprotein D (gD) of herpes simplex virus‐1 (HSV‐1). In contrast, 3‐OST5 exhibits broad substrate specificity and generate both HS‐binding motifs 5, 6, 7, 8, 9.

Although the reaction of 3‐O‐sulfation can be catalysed by the largest family of HS sulfotransferases, it is the least abundant modification in HS, and to date, very few biological mechanisms have been reported to be dependent on 3‐*O*‐sulfated HS 10. HSV‐1 gD was the first protein described as a specific ligand for highly sulfated HS motifs containing 3‐*O*‐sulfated GlcN residue. In addition to 3‐*O*‐sulfated HS, HSV‐1 gD was also reported to interact with other receptors, including nectin‐1 and ‐2, two cellular adhesion molecules of the immunoglobulin superfamily, and herpes virus entry mediator (HVEM), which belongs to the TNF‐α receptor superfamily 5, 11. Depending on the cell type, one or more receptors are critically required for the virus entry into host cells. For example, interaction between HSV‐1 gD and 3‐*O*‐sulfated HS is necessary to promote the membrane fusion process allowing the virus to enter into fibroblasts and haematopoietic cells, even though HVEM and nectin‐2 are also present at cell surface 5, 12, 13. In contrast, the presence of nectin‐1 is sufficient to promote membrane fusion and HSV‐1 entry into epithelial and neuronal cells 14, 15, 16. In addition to its role in the fusion between viral envelope and host cell membrane, HSV‐1 gD also acts as a signalling molecule and conditions host cells for viral replication. Thus, HSV‐1 gD has been shown to trigger the activation of nuclear factor‐kappa B (NF‐κB), which participates in the protection of the myeloid U937 cells against apoptosis 17, 18.

In previous studies, we demonstrated that cyclophilin B (CyPB) is an inflammatory factor, which triggers migration and integrin‐mediated adhesion of T‐lymphocytes and monocytes/macrophages via interactions with two types of binding sites, CD147 and cell surface HS 19, 20, 21. Importantly, we found that functional binding of CyPB was dependent on the interaction with 3‐*O*‐sulfated HS. Indeed, silencing the expression of 3‐OST3B strongly reduced the responses in T‐lymphocytes, thus confirming that 3‐O‐sulfation is a key modification that provides specialized HS structures for CyPB binding 22. We also demonstrated that the minimal heparin motif for CyPB binding is an octasaccharide, which contains a 3‐*O*‐sulfated GlcNH_2_ 23, 24. Interestingly, such structural features had been described in the heparin binding motif for HSV‐1 gD 25, 26. Moreover, HS 3‐O‐sulfation by 3‐OST3B was also reported to provide binding sites for HSV‐1 5, thus suggesting that HS motifs with binding properties for HSV‐1 gD and CyPB could be the same.

A soluble form of HSV‐1 gD was reported to protect myeloid cells against apoptosis 17, 18. In a recent work, we demonstrated that CyPB was capable of attenuating proinflammatory response in primary macrophages 27. These findings prompted us to investigate whether both 3‐*O*‐sulfated ligands could share the same antiapoptotic activity towards macrophages. First, we checked that soluble HSV‐1 gD was efficient to trigger cellular responses in human primary macrophages. We then analysed whether HSV‐1 gD and CyPB were capable of protecting macrophages against staurosporin‐induced apoptosis. After demonstrating that these cells mainly express HVEM, nectin‐2 and 3‐OST2 as 3‐*O*‐sulfated HS‐generating enzyme, we analysed their participation in the antiapoptotic activity of HSV‐1 gD and CyPB by RNA interference. We found that both 3‐*O*‐sulfated HS ligands induced the same responses in macrophages, by a common mechanism involving 3‐OST2 and HVEM. Collectively, our results suggest that HSV‐1 might hijack the binding sites for CyPB in order to protect host cells against apoptosis for efficient infection.

## Results

### Functional interactions of HSV‐1 gD with human primary macrophages

**Figure 1:**
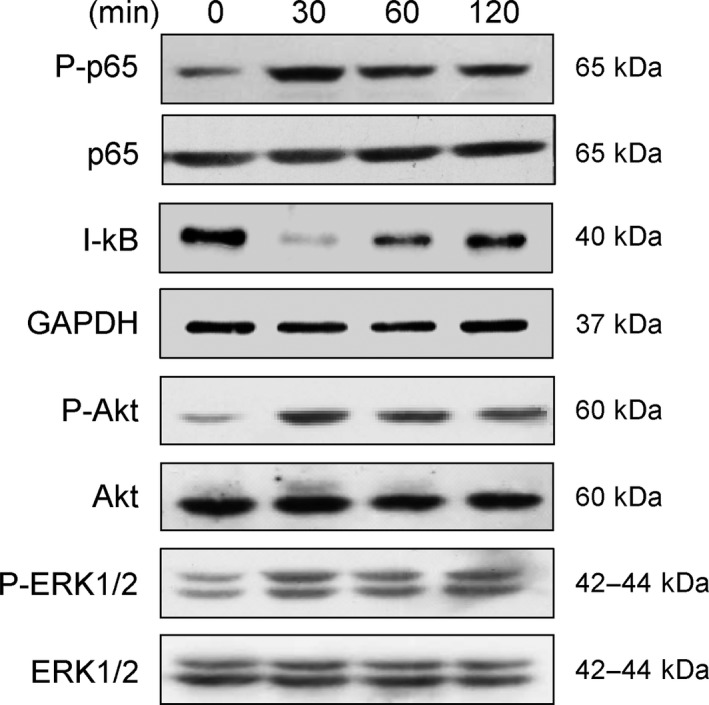
HSV‐1 gD‐induced signalling in human primary macrophages. Monocyte‐derived macrophages were stimulated with HSV‐1 gD (1 μg·mL^−1^) for the indicated times. Following cell lysis, proteins were separated by SDS/PAGE and subjected to western blotting with antibodies to I‐κB, phospho‐NF‐κB p65 (P‐p65), phospho‐ERK1/2 (P‐ERK1/2) and phospho‐Akt (P‐Akt). Parallel immunoblotting with antibodies to NF‐κB p65 subunit, GAPDH, ERK1/2 and Akt confirmed equal loading of samples. Data are representative of three separate experiments conducted with cells from distinct donors.

In their previous works, Sciortino *et al*. 18 had reported that a soluble form of HSV‐1 gD was capable of activating NF‐κB pathway in U937 myeloid cells. In the current study, we intended to confirm these findings with human primary macrophages and to investigate whether HSV‐1 gD was also efficient to activate extracellular signal‐regulated kinases (ERK) 1/2 and Akt kinases. Exposure of macrophages to 1 μg·mL^−1^ of a recombinant form of HSV‐1 gD (25 nm) led to a rapid degradation of I‐κB. Concomitantly, we observed an increase in the phosphorylation of NF‐κB p65 subunit, which confirmed that NF‐κB was efficiently activated following its dissociation from its sequestrating inhibitor. In addition, we found that ERK1/2 and Akt were rapidly phosphorylated following exposure to HSV‐1 gD. A time‐course analysis revealed that the activation of the kinases was maximal at 30 min poststimulation and extended over 2 h of stimulation (Fig. 1).

**Figure 2:**
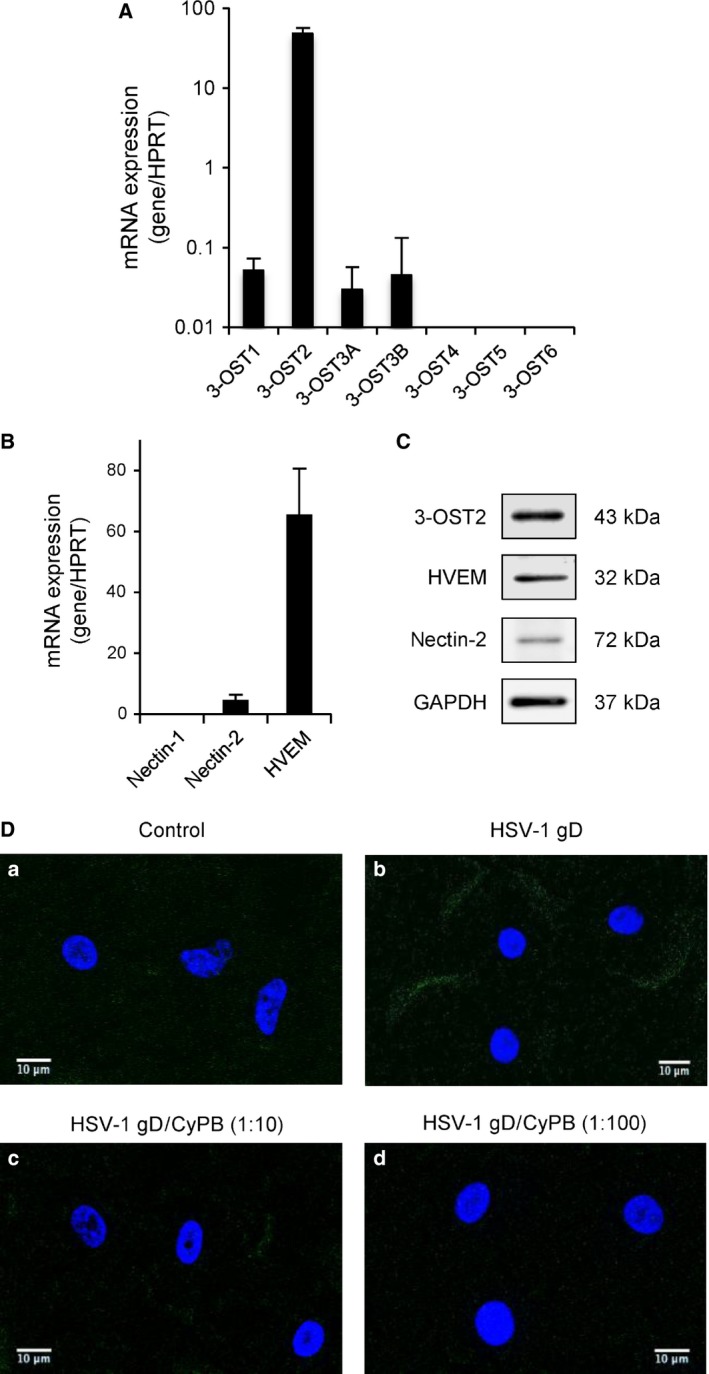
Identification of the HSV‐1 gD binding sites in primary macrophages. Analysis of the expression of mRNA encoding 3‐*O*‐sulfated HS‐generating enzymes (A) and HSV‐gD receptors (B). Following reverse transcription of RNA extracted from primary macrophages, the level of mRNA for 3‐OSTs, HVEM, nectin‐1 and nectin‐2 was quantified by real‐time RT‐PCR. Relative transcript abundance was normalized to HPRT mRNA. Data are means ± SD and were obtained with macrophages from six different donors. (C) Immunostaining of endogenous HVEM, nectin‐2 and 3‐OST2 in macrophages. Macrophages from the same donor were lysed and the expression of proteins of interest was analysed by western‐blot. Detection of GAPDH confirmed equal loading of samples. Representative results from three separate experiments are shown. (D) Competitive experiments for HSV‐1 gD binding to macrophages. Cells were incubated with 10 μg·mL^−1^ (250 nm) of His‐tagged gD in the absence (panel b) or presence of CyPB at 2.5 μm (panel c) or 25 μm (panel d). After 1 h of incubation at 4 °C, HSV‐1 gD binding was detected with an anti‐His‐tag antibody conjugated to Alexa 488 for analysis by confocal microscopy (green fluorescence). Control (panel a) was determined in the absence of any ligand. DAPI staining allowed visualization of cell nuclei (blue fluorescence). Scale bar = 10 μm. Images were representative of five experiments conducted with cells from distinct donors.

HSV‐1 gD is capable of interacting with various cell types, via its surface binding to 3‐*O*‐sulfated HS and/or to one among the three receptors nectin‐1, nectin‐2 and HVEM. In an attempt to identify the mechanisms by which HSV‐1 gD initiated signalling events in macrophages, we decided to analyse the expression of these binding sites by real‐time reverse transcription‐polymerase chain reaction (RT‐PCR). First, we confirmed that 3‐OST2 was the main 3‐*O*‐sulfated HS‐generating enzyme expressed in macrophages 28. In contrast, 3‐OST1, 3A and 3B were weakly expressed, and 3‐OST4, 5 and 6 were not detected (Fig. 2A). We also found that macrophages expressed a very high level of mRNA encoding HVEM. By comparison, nectin‐2 was poorly expressed and the level of nectin‐1 mRNA was barely detectable (Fig. 2B). The expression of 3‐OST2, HVEM and nectin‐2 in macrophages was confirmed by western blot (Fig. 2C). As expected, we found a high expression of HVEM, while nectin‐2 was less represented. In addition, 3‐OST2 was strongly expressed in macrophages, suggesting that it could efficiently participate to the synthesis of 3‐*O‐*sulfated HS with binding properties for HSV‐1 gD 8.

In previous works, we demonstrated that CyPB was also a ligand of 3‐*O*‐sulfated HS, for which the synthesis was dependent on the activity of type gD 3‐OSTs 24. Thus, we hypothesized that CyPB could compete with the binding of HSV‐1 gD to macrophages. To this end, we used a recombinant His‐tagged HSV‐1 gD protein, for which the binding was detected with an Alexa‐488 anti‐His‐tag antibody 29. In our hands, a nonspecific scattered staining of macrophages was observed with primary antibody alone (Fig. 2D, panel a). In contrast, incubation with 250 nm HSV‐1 gD (10 μg·mL^−1^) prior to the addition of Alexa‐488 antibody led to an additional fluorescent staining located at the cell membrane of macrophages (Fig. 2D, panel b). A significant staining was already observed in the presence of 25 nm of HSV‐1 gD (data not shown). However, the intensity of the fluorescence signal was stronger at 250 nm, which suggests that the highest concentration was required to maintain a full saturation of cell surface binding sites. Surface staining with HSV‐1 gD was partially reduced in the presence of a 10‐fold molar excess of CyPB (Fig. 2D, panel c) and completely abolished with a 100‐fold molar excess of CyPB (Fig. 2D, panel d). These observations thus support the idea that both proteins probably shared common binding sites at the surface of macrophages. Interestingly, we previously reported that CyPB was capable of activating ERK1/2, Akt and NF‐κB in macrophages to a similar extent than HSV‐1 gD 27. Thus, these observations suggest that both proteins could also trigger similar responses in macrophages.

### HSV‐1 gD and CyPB‐mediated protection of macrophages against apoptosis

**Figure 3:**
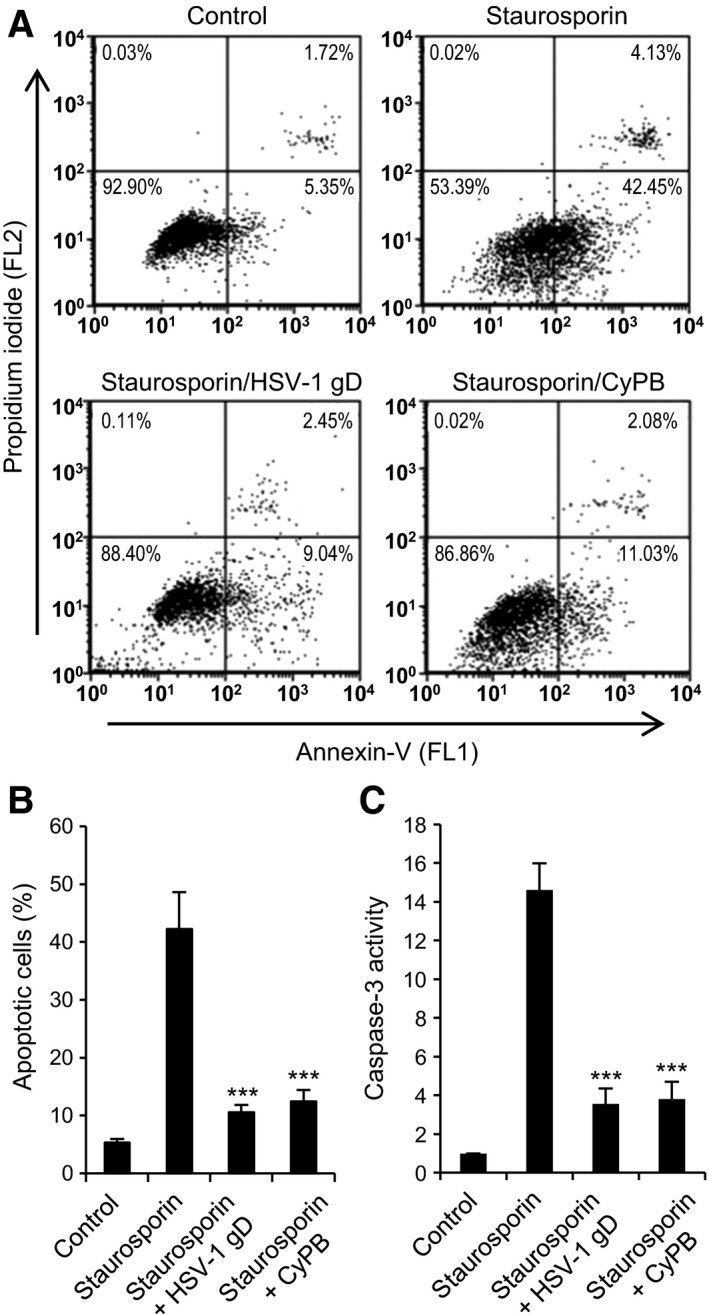
Protective effects of HSV‐1 gD and CyPB against apoptosis in primary macrophages. (A, B) Flow cytometry analysis of staurosporin‐induced apoptosis. Macrophages were either untreated or treated with HSV‐1 gD or CyPB (both at 1 μg·mL^−1^) for 8 h and subsequently exposed to staurosporin (0.5 μm) for 4 h. At the end of incubation, cells were stained with fluorescein‐conjugated annexin‐V (FL1) and PI (FL2) for flow cytometry analysis. (A) Representative dot‐blots showing the distribution of viable (annexin‐V^−^/PI^−^), early apoptotic (annexin‐V^+^/PI^−^), late apoptotic (annexin‐V^+^/PI^+^) and necrotic (annexin‐V^−^/PI^+^) cell populations. (B) Percentages of early apoptotic cells (annexin‐V^+^/PI^−^). Values are means ± SD from five experiments conducted with macrophages from distinct donors. (C) Analysis of caspase‐3 activation. Following incubation in the absence or presence of HSV‐1 gD or CyPB, macrophages were exposed to staurosporin for 4 h, after which they were lysed. Caspase‐3 activity was then measured in cell lysates using the fluorescent Ac‐DEVD‐AMC substrate, as described in “Materials and methods”. Data are expressed as fold increase in caspase‐3 activity by comparison with cells cultured in the absence of staurosporin. Results are means ± SD of five independent experiments performed with cells isolated from distinct donors (***P < 0.001, significantly different when compared to cells exposed to staurosporin alone).

In their previous works, Medici *et al*. 17 reported that HSV‐1 was capable of protecting U937 cells against apoptosis. They also demonstrated that cell treatment with recombinant HSV‐1 gD was efficient enough to reproduce the antiapoptotic property of the virus. Hence, we investigated whether HSV‐1 gD was capable of inducing a similar response in primary macrophages. To this end, monocyte‐derived macrophages were incubated with HSV‐1 gD (25 nm) for 8 h, after which apoptosis was induced by the addition of 0.5 μm staurosporin. We decided to use this proapoptotic drug because of its efficiency to induce a full activation of caspase‐3 in primary macrophages 18, 30, 31. The experimental conditions for induction of apoptosis by staurosporin were retained to reduce necrosing effects of the proapoptotic drug in primary macrophages. In first experiments, cell apoptosis was evaluated by analysing phosphatidylserine externalization, a mechanism that reflects the earlier stages of apoptosis. Following treatment with staurosporin, macrophages were stained with fluorescent annexin‐V and propidium iodide (PI) and analysed by flow cytometry (Fig. 3A,B). In the absence of any treatment, the percentage of apoptosis (including early and late apoptotic cells) was < 8%. As expected, cell treatment with staurosporin resulted in a strong increase in apoptosis, with a number of early apoptotic cells corresponding to 42 ± 6% of the whole cell population. Moreover, the percentages of late apoptotic and necrotic cells were less than 5% and 0.5%, respectively, confirming that our experimental conditions were appropriate to measure early events of apoptosis. Exposure of macrophages to HSV‐1 gD prior to the treatment with staurosporin reduced the percentage of early apoptotic cells to 10.5 ± 2%, thus confirming the protective property of the viral protein 17. We then reproduced the same experiment with CyPB. To a similar extent, macrophages were exposed to CyPB (50 nm) for 8 h, after which apoptosis was induced by the addition of staurosporin for 4 h. Under these conditions, the percentage of early apoptotic cells was at 12.4 ± 3%, which was close to the value obtained with HSV‐1 gD. In order to validate these first results, we next examined the inhibitory property of both proteins on the activation of caspase‐3, because of the key role of this protease in the induction of apoptosis. As shown in Fig. 3C, we found that cell treatment with staurosporin strongly increased the activity of caspase‐3 (×14.5 when compared with untreated cells). Exposure of macrophages to either HSV‐1 gD or CyPB prior to the addition of staurosporin was effective to reduce the activation of caspase‐3 to a similar extent. Indeed, the activity of caspase‐3 was decreased by 75% in cells exposed to both proteins, when compared to the response measured in cells treated with staurosporin alone. Collectively, these data indicate that HSV‐1 gD and CyPB shared similar protective properties against apoptosis induced by staurosporin in primary macrophages.

### Effect of HSV‐1 gD and CyPB on the induction of antiapoptotic genes in macrophages

**Figure 4:**
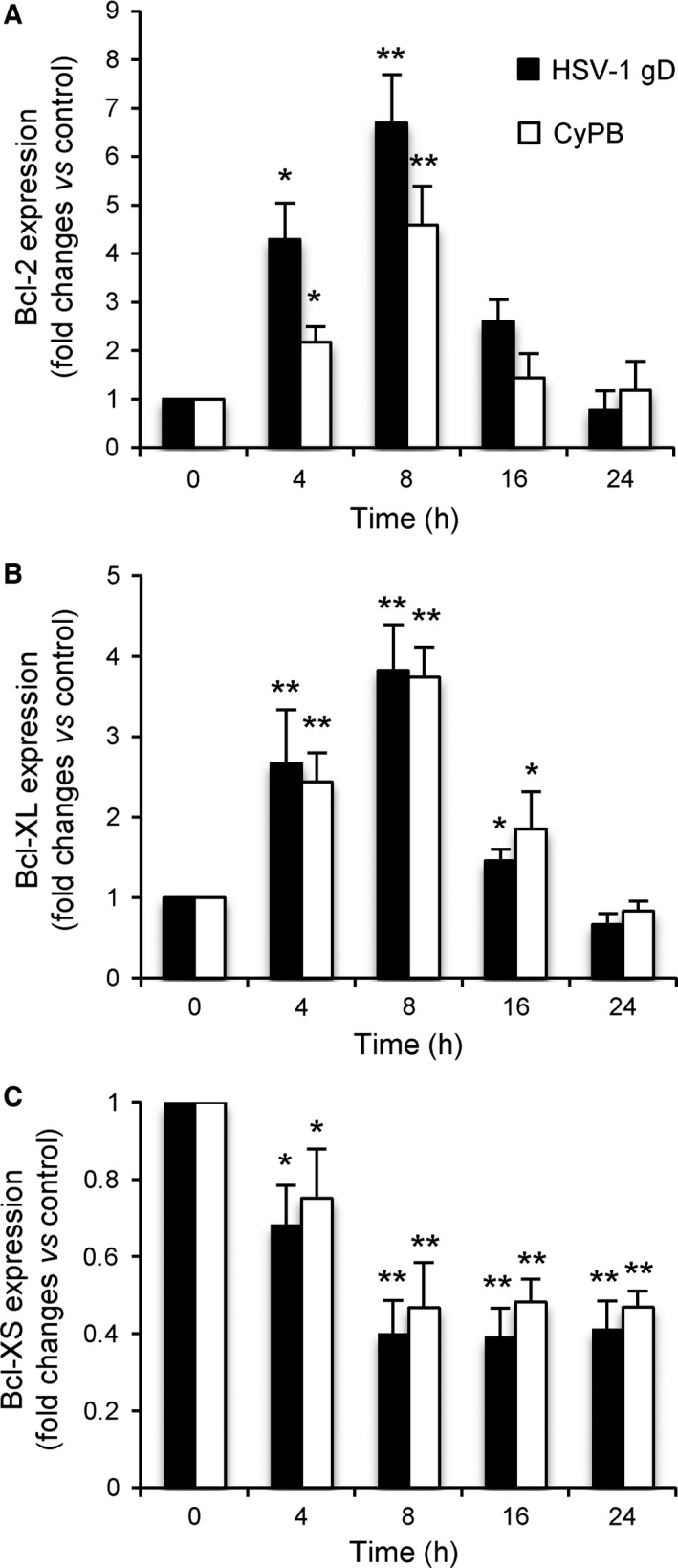
Modulation of the expression of apoptotic genes in primary macrophages. Macrophages were incubated in the presence of HSV‐1 gD or CyPB, both at 1 μg·mL^−1^. At the indicated times, cells were harvested and the expression of mRNA encoding Bcl‐2, Bcl‐XL and Bcl‐XS was analysed by real‐time RT‐PCR. Relative transcript abundance was normalized to endogenous HPRT mRNA. Results are expressed as fold changes by comparison with nonstimulated cells. Values correspond to means ± SD of five independent experiments conducted with macrophages from distinct donors (*P < 0.05, **P < 0.01, significantly different when compared to unstimulated cells).

Previous works reported that the expression of *Bcl‐2* and *Bcl‐2L1* genes was upregulated in human fibroblasts exposed to HSV‐1. Interestingly, this response was no more observed with ΔHSV‐1 gD virions, thus illustrating a critical role of HSV‐1 gD in the mechanisms leading to *Bcl‐2* and *Bcl‐2L1* overexpression 32. Depending on cell environment, two proteins with antagonist functions can be produced from *Bcl‐2L1* gene by mRNA splicing: the longer form, termed B‐cell lymphoma (Bcl)‐XL, exhibits antiapoptotic activity, while the shorter form, termed Bcl‐XS, is a promoter of apoptosis 33. Hence, we analysed the ability of HSV‐1 gD and CyPB to modulate the expression of mRNA encoding Bcl‐2, Bcl‐XL and Bcl‐XS in macrophages using real‐time RT‐PCR (Fig. 4). Time‐course experiments revealed that the levels of Bcl‐2 and Bcl‐XL mRNAs were similarly increased in response to either HSV‐1 gD or CyPB, with an expression peaking at 8 h poststimulation. Concomitantly, the level of mRNA encoding Bcl‐XS decreased in cells exposed to HSV‐1 gD or CyPB. The inhibitory effect (~ 50%) was maximal at 8 h poststimulation and was maintained over 24 h. Taken together, these results indicate that HSV‐1 gD and CyPB are capable of regulating the balance between pro‐ and antiapoptotic factors, which is consistent with the protective properties of these proteins against apoptosis.

### Silencing of the expression of 3‐OST2 and HSV‐1 gD receptors by RNA interference

**Figure 5:**
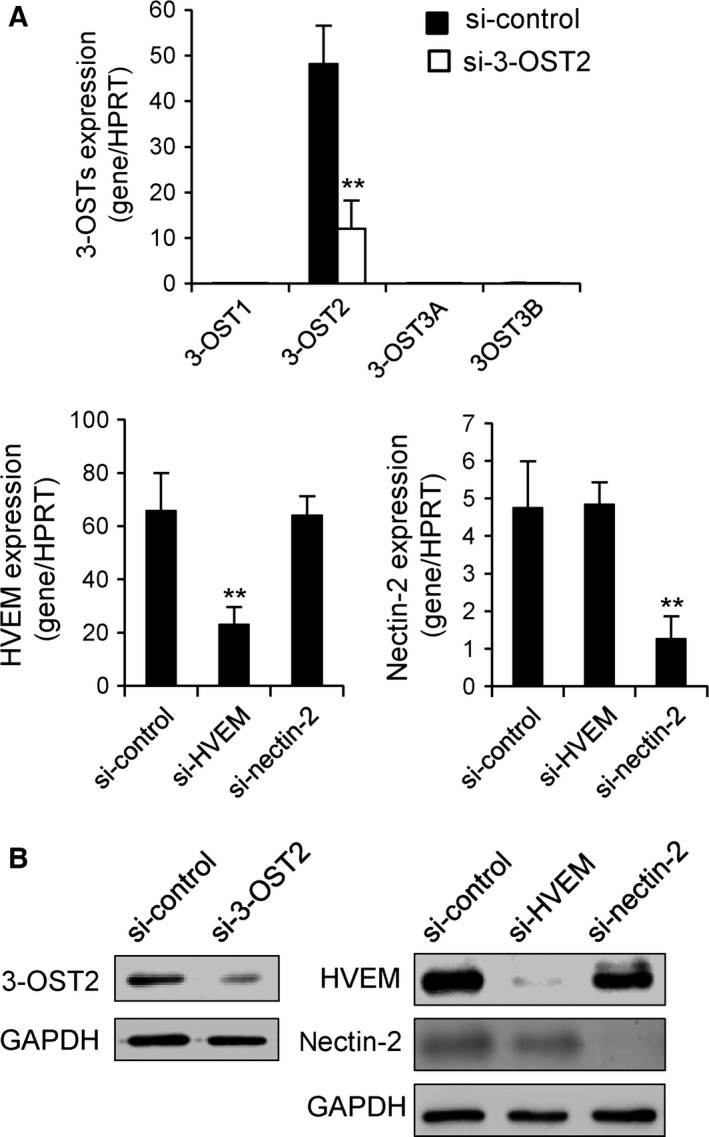
Downregulation of the expression of nectin‐2, HVEM or 3‐OST2 by RNA interference. Synthetic siRNA (termed si‐3‐OST2, si‐HVEM and si‐nectin‐2) were used to specifically inhibit the expression of 3‐OST2, HVEM and nectin‐2 in human macrophages. Following transfection of macrophages with si‐3‐OST2, si‐HVEM and si‐nectin‐2, the expression of mRNAs encoding 3‐OSTs, HVEM and nectin‐2 was quantified by real‐time RT‐PCR (A). A negative control siRNA (si‐control) was used to check for the specificity of silencing. Relative transcript abundance was normalized to HPRT mRNA. Data are means ± SD of five independent experiments conducted with macrophages from distinct donors (**P < 0.01, significantly different when compared to cells transfected with si‐control). (B) The efficacy of siRNA to downregulate the expression of 3‐OST2, HVEM and nectin‐2 was checked by western blot. Parallel immunoblotting with anti‐GAPDH confirmed equal loading of the samples. Representative results from three separate experiments are shown.

In order to decipher the underlying mechanisms responsible for the responses induced by HSV‐1 gD and CyPB in macrophages, we used an approach based on RNA interference. We focused our interest on 3‐OST2, HVEM and nectin‐2, because of their higher expression when compared to other 3‐OST isoenzymes and nectin‐1. Treatment of macrophages with specific small‐interfering RNA (siRNA) targeting 3‐OST2, HVEM and nectin‐2 (termed si‐3‐OST2, si‐HVEM and si‐nectin‐2, respectively) resulted in a significant downregulation of corresponding mRNA (Fig. 5A). After 48 h of transfection, the inhibitory effects were at 75%, 72% and 74%, respectively, when compared to the results obtained with control siRNA. Importantly, we checked that the levels of mRNA encoding 3‐OST1, 3‐OST3A and 3‐OST3B were not modified in the presence of si‐3‐OST2. Similarly, si‐HVEM and si‐nectin‐2 significantly reduced the expression of their target mRNA, without any cross‐reaction. The efficiency of each siRNA was then confirmed by analysing the production of 3‐OST2, HVEM and nectin‐2 in cell lysates by western blot (Fig. 5B). As expected, we found that the levels of expression of 3‐OST2, HVEM and nectin‐2 were considerably reduced in macrophages treated with specific siRNA for 48 h. In addition, no significant change in the expression of HVEM was observed in macrophages treated with siRNA targeting nectin‐2 and vice versa, thus validating the use of these siRNA for our next experiments.

### Identification of functional binding sites for HSV‐1 gD and CyPB

**Figure 6:**
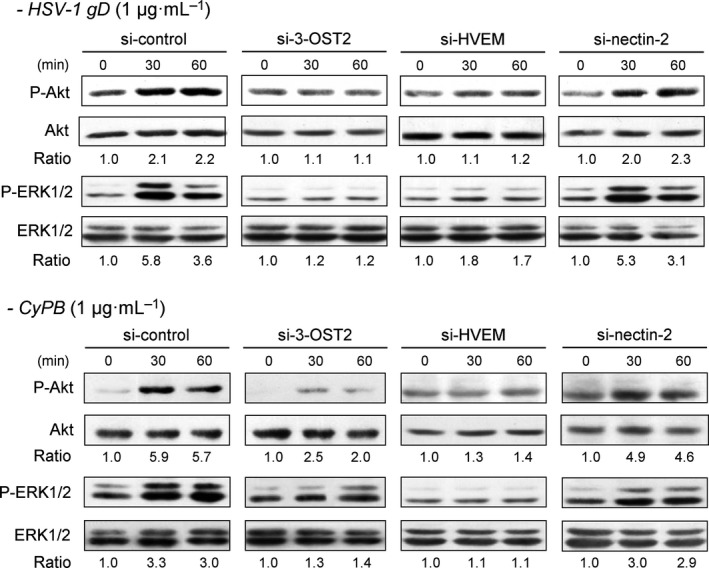
Effect of silencing the expression of 3‐OST2, HVEM and nectin‐2 on HSV‐1 gD‐ and CyPB‐induced signalling in macrophages. The contribution of 3‐OST2 (as a 3‐*O*‐sulfated HS‐generating enzyme) and of HVEM and nectin‐2 to the activation of signalling pathways was evaluated by analysing the phosphorylation of Akt and ERK1/2 in siRNA‐transfected macrophages. Following treatment with siRNA for 48 h, cells were stimulated with HSV‐1 gD or CyPB (both at 1 μg·mL^−1^) for various times, and the phosphorylation of ERK1/2 (P‐ERK1/2) and Akt (P‐Akt) was analysed by western blot. Parallel immunoblotting with antibodies to Akt and ERK1/2 confirmed equal loading of samples. Data are representative of three independent experiments. The ratio of P‐Akt/Akt and P‐ERK1/2/ERK1/2 were quantified and normalized to unstimulated cells.

In order to know whether the signalling events induced by HSV‐1 gD and CyPB are dependent on the interactions with HVEM, nectin‐2 and/or 3‐*O*‐sulfated HS, we decided to analyse the activation of ERK1/2 and Akt in siRNA‐treated macrophages (Fig. 6). As expected, cell treatment with the negative control siRNA did not hamper the responses induced by either HSV‐1 gD or CyPB. Both stimuli were still efficient to induce the phosphorylation of ERK1/2 and Akt in macrophages, with a maximal activation observed at 30 min of stimulation. The same experiments were then reproduced with specific siRNAs. When compared to control cells, we found that silencing the expression of nectin‐2 did not significantly alter the activation of ERK1/2 and Akt, thus indicating that this receptor was not involved in the responses induced by HSV‐1 gD and CyPB. In contrast, downregulation of 3‐OST2 strongly reduced the ability of both proteins to activate Akt and ERK1/2. These results were, however expected, because of the requirement of 3‐*O*‐sulfated HS in the binding of HSV‐1 gD and CyPB to responsive cells. When analysing the phosphorylation status of ERK1/2 and Akt in macrophages treated with si‐HVEM, we found a similar inhibitory effect on the responses induced by both stimuli. Although the results obtained with HSV‐1 gD were in agreement with the literature data, they were unexpected for CyPB. Indeed, HVEM has been well‐described as a functional receptor for HSV‐1 gD 18, 34. In contrast, we are the first to demonstrate a functional interaction between HVEM and CyPB, which suggests that both proteins shared the same signalling receptor in macrophages. Collectively, these observations suggest that HSV‐1 gD and CyPB probably interact with a signalling complex formed by the association of 3‐*O*‐sulfated HS and HVEM at the surface of macrophages.

### Role of HVEM and 3‐*O*‐sulfated HS in the antiapoptotic activity of HSV‐1 gD and CyPB

**Figure 7:**
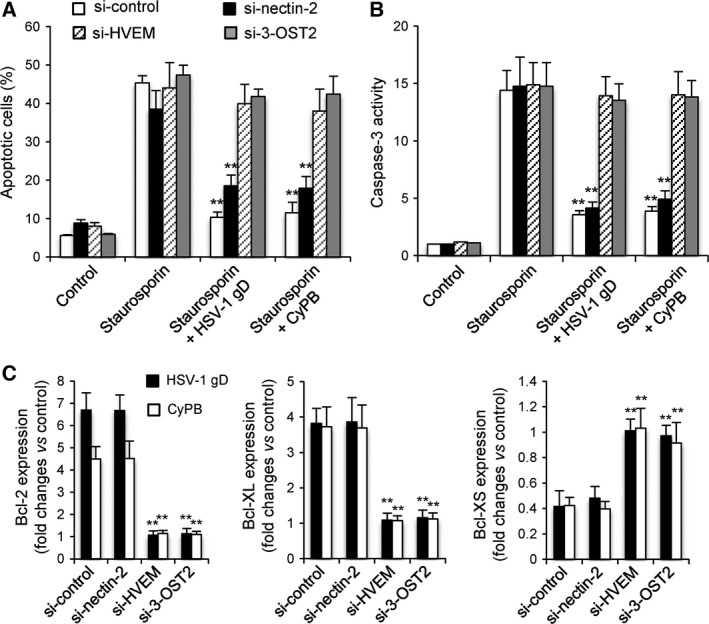
Effects of silencing the expression of 3‐OST2, HVEM and nectin‐2 on the anti‐apoptotic properties of HSV‐1 gD and CyPB. The contribution of 3‐OST2 (as a 3‐*O*‐sulfated HS‐generating enzyme) and of HVEM and nectin‐2 to the protective effect of HSV‐1 gD and CyPB was analysed by measuring staurosporin‐induced apoptosis in siRNA‐treated macrophages. Following treatment with siRNA for 48 h, cells were incubated or not with HSV‐1 gD and CyPB (1 μg·mL^−1^) for 8 h, after which they were exposed to staurosporin (0.5 μm) for 4 h. (A) The percentage of apoptotic cell population was evaluated by analysing the binding of fluorescein‐conjugated annexin‐V. Each bar of histogram represents mean ± SD of the rate of apoptotic cells (annexin‐V^+^/PI^−^) obtained from five distinct experiments. (B) In parallel experiments, the activation of caspase‐3 was analysed in siRNA‐treated cells after exposure to staurosporin. Results are expressed as fold increase in caspase‐3 activity by comparison with untreated cells and correspond to means ± SD from five independent experiments (**P < 0.01, significantly different when compared with the results obtained with staurosporin alone). (C) Variations in the expression of Bcl‐2, Bcl‐XL and Bcl‐XS was analysed in siRNA‐treated macrophages following incubation with HSV‐1 gD or CyPB (1 μg·mL^−1^) for 8 h. After extraction of total RNA and reverse transcription, the expression of mRNA encoding Bcl‐2, Bcl‐XL and Bcl‐XS was analysed by real‐time RT‐PCR. Relative transcript abundance was normalized to HPRT mRNA. Results are expressed as fold changes by comparison with nonstimulated cells and correspond to means ± SD of three separate experiments (**P < 0.01, significantly different when compared to the results obtained with negative control siRNA).

To gain evidence into the relationships between the responses induced by HSV‐1 gD and CyPB in macrophages and the interactions of both proteins with 3‐*O*‐sulfated HS and HVEM, we analysed the antiapoptotic properties of both proteins in siRNA‐treated cells. As expected, treatment of macrophages with negative control siRNA did not alter the protective effect of HSV‐1 gD and CyPB against apoptosis, as demonstrated by their efficiency to reduce annexin‐V binding (Fig. 7A) and caspase‐3 activation (Fig. 7B) in response to staurosporin. Similarly, silencing the expression of nectin‐2 had no notable effect on the capability of HSV‐1 gD and CyPB to inhibit staurosporin‐induced apoptosis, which confirmed that the responses induced by both proteins were not dependent on the expression of this receptor. In contrast, downregulation of HVEM and 3‐OST2 rendered the cells unresponsive to both stimuli, thus restoring the capacity of staurosporin to induce apoptosis. Indeed, the rates of annexin‐V binding and caspase‐3 activation were not significantly modified by the addition of HSV‐1 gD and CyPB, when compared to the same siRNA‐treated cells exposed to staurosporin alone. Finally, we analysed the expression of Bcl‐2, Bcl‐XL and Bcl‐XS in siRNA‐treated macrophages exposed to HSV‐1 gD and CyPB (Fig. 7C). First, we found that cell treatment with negative control siRNA or si‐nectin‐2 did not significantly modify the expression of the genes encoding Bcl‐2, Bcl‐XL and Bcl‐XS in response to both proteins. As previously shown in Fig. 4, HSV‐1 gD and CyPB were efficient to increase the mRNA levels of Bcl‐2 and Bcl‐XL, which was associated to a decrease in the expression of Bcl‐XS. In contrast, cell treatment with si‐HVEM or si‐3‐OST2 maintained the mRNA levels of Bcl‐2, Bcl‐XL and Bcl‐XS to basal level, confirming that silencing the expression of HVEM and 3‐OST2 rendered macrophages unresponsive to the protective effect of HSV‐1 gD and CyPB.

In previous works, CyPB was reported to induce migration and integrin‐mediated adhesion of T‐lymphocytes and monocytes/macrophages, by a mechanism dependent on CD147 19, 20, 21, 35, 36, 37. Thus, we analysed the antiapoptotic responses triggered by CyPB and HSV‐1 gD in macrophages that were pretreated with siRNA targeting CD147. In our experiments, CD147 expression was reduced by < 80%, because stronger inhibition altered the viability of macrophages. We found that the protective effect of CyPB was not modified in si‐CD147‐treated cells. However, a plausible explanation might be that downregulation of CD147 expression was not sufficient to visualize an effect on the response of CyPB. Nevertheless, we found that HSV‐1 gD was as efficient as CyPB to prevent staurosporin‐induced apoptosis and to induce the expression of antiapoptotic proteins in si‐CD147‐treated cells (data not shown). Thus, both proteins retained their antiapoptotic activity in si‐CD147‐treated cells, which suggests that CD147 is not involved in the antiapoptotic activity of CyPB.

Taken together, these results support the idea that HSV‐1 gD and CyPB shared the same protective activity in macrophages by interacting with a complex formed by the association of HVEM and 3‐*O*‐sulfated HS.

## Discussion

Due to their high structural heterogeneity, HS are capable of interacting with numerous extracellular mediators, such as growth factors, morphogens, cytokines, chemokines, adhesion molecules or viral glycoproteins, for which they control bioavailability and functions. Consequently, HS are involved in many physiological and pathological processes, including cellular proliferation, differentiation, adhesion, migration and viral infection 1, 2, 3, 4. The structural distinction in HS sequences is derived from enzymatic modifications during the maturation phase of the glycanic backbone in the Golgi apparatus. Although the reaction of 3‐O‐sulfation is catalysed by the largest family of HS sulfotransferases, it is the least abundant modification and to date, very few proteins have been described as ligands for 3‐O‐sulfated HS 10. Among them, HSV‐1 gD and CyPB have been the subject of numerous studies showing that their respective activities were dependent on the interactions with HS motifs containing a 3‐*O*‐sulfated GlcN residue 5, 6, 7, 8, 9, 24. In addition to control the critical step of membrane fusion between the virus and its target cells, HSV‐1 gD was also reported as the main signalling molecule within HSV‐1 envelope to condition host cells for viral replication. A soluble form of HSV‐1 gD was as efficient as the viral particle to protect myeloid cells against apoptosis. Moreover, HSV‐1 gD was capable of modulating the expression of apoptosis‐related genes in primary fibroblasts, thus leading to an increase in cell survival 17, 18, 32. However, we demonstrated that CyPB was effective in reducing proinflammatory response in human primary macrophages 27. In this context, we decided to test the hypothesis that HSV‐1 gD and CyPB could trigger common responses in monocyte‐derived macrophages.

First, we confirmed that exposure of primary macrophages to a soluble form of HSV‐1 gD led to the activation of NF‐κB, Akt and ERK1/2 signalling molecules and to the protection against staurosporin‐induced apoptosis. Previous studies reporting a protective effect of HSV‐1 gD had been undertaken in U937 cells with anti‐Fas antibody and staurosporin as apoptosis inducers 17, 18. However, primary macrophages are known to be resistant to Fas‐induced apoptosis 31. Thus, we decided to use staurosporin to obtain a full activation of proapoptotic pathways in monocyte‐derived macrophages 30. In our experimental model, the protective effects of HSV‐1 gD were accompanied by upregulation of genes encoding the antiapoptotic Bcl‐2 and Bcl‐XL and concomitant decrease in the expression of proapoptotic Bcl‐XS, which is consistent with previous results obtained in myeloid cells and fibroblasts. In parallel experiments, we found that CyPB triggered the same antiapoptotic responses in macrophages. In our previous works, we had demonstrated that CyPB also triggered the activation of NF‐κB, Akt and ERK1/2 in macrophages 27. Hence, the current results extend our previous studies and demonstrate that, besides promigratory and anti‐inflammatory activities 19, 27, CyPB may induce similar protective effects as HSV‐1 gD against apoptosis in human macrophages.

Following its attachment to the surface of target cells, HSV‐1 entry is dependent on the fusion between the viral envelope and host cell membrane, a mechanism requiring the participation of HSV‐1 gD. In addition to 3‐*O*‐sulfated HS, other cell surface binding sites for HSV‐1 gD have been described, i.e. HVEM, nectin‐1 and nectin‐2. The relative contribution of these diverse HSV‐1 gD binding sites allows the virus to infect multiple target cells. As example, HSV‐1 entry in fibroblasts and hematopoietic cells is critically dependent on 3‐*O*‐sulfated HS, even though HVEM and nectin‐2 are also present at the cell surface 5, 12, 13. In contrast, nectin‐1 is sufficient for HSV‐1 entry within epithelial and neuronal cells 14, 15, 16. We then analysed the expression of HSV‐1 receptors and 3‐*O*‐sulfated HS‐generating enzymes in primary macrophages. We found that HVEM was the major receptor present in these cells, while nectin‐2 was also detected but to a lower level of expression. In accordance with our previous results, we also confirmed that 3‐OST2 is the main 3‐OST isoenzyme expressed in macrophages 28. In order to identify the way by which HSV‐1 gD induced protective responses in macrophages, we used an approach based on RNA interference. We found that silencing the expression of 3‐OST2 strongly reduced the ability of HSV‐1 gD and CyPB to induce the activation of Akt and ERK1/2 and to modulate the expression of Bcl‐2, Bcl‐XL and Bcl‐XS. Moreover, the protective effect of both proteins against staurosporin‐induced apoptosis was considerably altered. Thus, these results confirmed that the responses induced by HSV‐1 gD and CyPB are dependent on the interactions with 3‐*O*‐sulfated HS. Given that 3‐OST2 is highly expressed in primary macrophages, it is likely to contribute to the generation of the majority of 3‐*O*‐sulfated HS species. Notably, we demonstrated that surface binding of HSV‐1 gD to macrophages was efficiently inhibited by an excess of CyPB. Thus, these results further support the idea that 3‐*O*‐sulfated HS motifs with binding properties for HSV‐1 gD and CyPB are probably the same. In parallel experiments, we found that silencing the expression of HVEM strongly reduced the responses induced by HSV‐1 gD and CyPB in macrophages, while downregulation of nectin‐2 had no notable inhibitory effect. These results indicate that HVEM participates in the activation of signalling pathways induced by HSV‐1 gD and contributes to its protective effect against apoptosis. However, it is important to note that HVEM and 3‐*O*‐sulfated HS have not redundant activities. Indeed, silencing the expression of 3‐OST‐2 was as efficient to obtain a full inhibition of the responses induced HSV‐1 gD, indicating that the absence of 3‐*O*‐sulfated HS was not compensated by the presence of HVEM, and vice versa. Thus, these results suggest a model in which HSV‐1 gD probably interact with a signalling complex formed by the association of 3‐*O*‐sulfated HS and HVEM at the surface of macrophages. Surprisingly, silencing the expression of HVEM also altered the responses induced by CyPB. These findings were unexpected, because HVEM was not described as a receptor for CyPB. On the other hand, we and others reported that CyPB triggered the migration and adhesion of immune cells, via a mechanism involving CD147 19, 20, 35, 37. This glycoprotein is expressed in many cell types and possesses a diverse range of functions, which rely on its ability to interact with a number of binding partners. It is widely expressed in human tumours and plays a central role in the progression of many cancers by regulating cell proliferation, apoptosis and migration 38. Inhibitory agents targeting either CD147 or CyPB activity reduced the migration of immune cells, which indicates that CD147–CyPB interaction is mainly involved in the inflammatory response 19, 36, 37. However, we did not observe any direct interaction with CyPB, suggesting that CD147 is rather a cosignalling molecule involved in the activation of migratory pathways. In particular, we demonstrated that it participates to the association of CD98 with β1 integrins, which was required to promote monocyte adhesion in response to CyPB 21, 39. In that context, we verified whether CD147 could be also involved in the antiapoptotic activity of CyPB. In our experiments, silencing the expression of CD147 did not significantly modify the protective effect of CyPB, which remained as efficient as HSV‐1 gD to prevent staurosporin‐induced apoptosis and to induce the expression of antiapoptotic proteins in macrophages. Thus, these results suggested that CD147 did not participate in the common antiapoptotic activity of CyPB and HSV‐1gD. Altogether, our findings demonstrate for the first time that HSV‐1 gD and CyPB share the same antiapoptotic activity by interacting with the same binding sites, i.e. HVEM and 3‐*O*‐sulfated HS, at the surface of macrophages.

In their recent work, Oh *et al*. 40 reported that overexpressed CyPB decreased MPP+‐induced oxidative stress and inhibited the activation of proapoptotic molecules in SH‐SY5Y neuroblastoma cells. In a previous study, the same authors had showed that CyPB also protected PC12 cells from beta‐amyloid‐induced neurotoxicity in an *in vitro* Alzheimer disease model 41. Collectively, these results suggested that CyPB could protect neuronal cells from apoptosis in some pathological disorders. In that context, the current work further supports the assumption that CyPB exhibits a protective activity, at least in cells of neuronal and myeloid lineages, and revealed that HSV‐1 gD possibly shares the same antiapoptotic properties by hijacking the receptors of CyPB. In addition to this protective activity, HSV‐1 gD and CyPB were reported to trigger other common properties. Indeed, both of them exhibit anti‐inflammatory activities. We showed that CyPB was capable to attenuate the responses of proinflammatory macrophages, via a mechanism dependent on the expression of Bcl‐3 27. On the other hand, HSV‐1 gD was described to mimic the interaction between HVEM and B‐ and T‐lymphocyte attenuator, the latter being a natural ligand of the receptor. Such an interaction induces a tolerogenic environment, which leads to alterations in the immune response 42. Further investigations are needed to know whether the anti‐inflammatory properties of HSV‐1 gD and CyPB are also dependent on the interaction with HVEM and 3‐*O*‐sulfated HS, and to prove that the protective effect of CyPB is relevant *in vivo*.

HSV‐1 has been incriminated in the occurrence of corneal lesions and neurological damages in immunocompromised individuals and neonates. Recurrent herpetic stromal keratitis may lead to severe vision impairments, making this infection as the main cause of induced blindness in developed countries. It is characterized by fibroblast proliferation and chronic inflammation, which is maintained by the recruitment and survival of activated macrophages at the site of the lesion 12, 43. The implication of 3‐*O*‐sulfated HS in the mechanisms controlling HSV‐1 entry has been extensively studied and described to be specific of HSV‐1 gD 5, 12, 44. Here, we demonstrated that the interaction between HSV‐1 gD and 3‐*O*‐sulfated HS was also of critical importance for inducing survival signals in macrophages. Thus, our results provide new information on the mechanism by which HSV‐1 gD may participate to the persistence of inflamed macrophages in herpetic corneal lesions. Entry of HSV serotypes into cells depends on the interactions between multiple viral glycoproteins and host receptors. HSV‐1 shares common receptors and pathways that are also used by HSV‐2. Thus, HVEM and nectin‐1 are utilized by the gD glycoprotein of both viruses. Cell surface HS are also recognized by other viral glycoproteins, namely gB and gC. However, only HSV‐1 gD is capable of specifically interacting with 3‐*O*‐sulfated HS. This unique property may explain the preferential tropism of HSV‐1 for host cells that express high levels of certain 3‐OSTs 11, 44, 45. In previous work, we reported that M2 macrophages contained almost twice more HS than M1 macrophages, with a relatively higher sulfation rate. To gain information on these structural modifications upon macrophage polarization, we analysed the expression of HS sulfotransferases and we found that the most dramatic changes were observed in the expression of 3‐OST isoenzymes. While 3‐OST2 was highly expressed in M2 macrophages, it was replaced by 3‐OST3B in M1 macrophages 28. Both isoforms catalyse the same reaction of 3‐O‐sulfation *in vitro* and their ectopic expression in CHO cells allowed HSV‐1 infection 5, 8. Thus, these results suggest that macrophages are capable of generating HS‐binding sites for HSV‐1 gD irrespective of their polarization status. However, we also demonstrated that 2‐OST was more expressed in M2 than in M1 subtypes. Interestingly, type gD 3‐OSTs preferentially modify GlcNS or GlcNH_2_ residues adjacent to an IdoUA2S residue 10, which indicates that M2 macrophages have the enzymatic machinery to produce more HS‐binding sites for HSV‐1 gD than M1 macrophages. Therefore, M2 macrophages could be more responsive to the protective effect of HSV‐1 gD, which may induce their accumulation in the herpetic lesion and maintain persistent infection.

In this context, drugs targeting the interaction between the viral glycoprotein and 3‐*O*‐sulfated HS may be helpful for the development of therapeutic agents for treating corneal inflammation. Potential drugs may mimic HS oligosaccharides, and a growing interest has focused on the use of recombinant sulfotransferases to synthesize HS‐derived molecules with therapeutic application 46. As example, using a 3‐*O*‐sulfated heparin octasaccharide was effective to inhibit HSV‐1 infection by blocking viral entry 47. On the other hand, blocking the protein‐binding domains of HS chains with synthetic peptides may be also a valuable approach to inhibit the interaction with HSV‐1. In this way, Tiwari *et al*. 48 identified anti‐HSV‐1 agents in two groups of peptides showing either alternating (G1) or clustered (G2) positive charges. Interestingly, G2 peptide isolated against 3‐*O*‐sulfated HS was found to display wider ability to inhibit HSV‐1 entry. In previous works, we demonstrated that the interaction between HS and CyPB involved the N‐terminal extension of the protein, which is unique among the members of the cyclophilin family. This positively charged peptide contains two amino acid clusters, i.e. ^14^YFD^16^ and ^3^KKK^5^, which are spatially arranged so that they act synergistically to form a binding site for an HS octasaccharide 22, 49. On the assumption that both proteins interact with the same 3‐*O*‐sulfated HS motif, a synthetic peptide mimicking the N‐terminal extension of CyPB may be effective to specifically inhibit the responses triggered by HSV‐1 gD in primary macrophages. This possibility is currently under investigation in our laboratory.

## Materials and methods

### Materials

Recombinant HSV‐1 gD protein was purchased from Fitzgerald (Acton, MA, USA). Recombinant human CyPB was produced in *Escherichia coli*. Following purification, the protein was detoxified on Detoxi‐gel Endotoxin Removing Gel (Thermo Scientific, Waltham, MA, USA) as described in Marcant *et al*. 27. The efficacy of LPS removal was checked using a cell‐based assay with HEK‐Blue™‐hTLR4 cell line (Invivogen, Toulouse, France). Mouse antibodies to ERK1/2 and to nectin‐2 and goat antibody to HVEM were purchased from Sigma‐Aldrich (St. Louis, MO, USA). Rabbit antibody to 3‐OST2 was from Thermo Scientific. Rabbit antibodies to phosphorylated ERK1/2 and to phosphorylated Akt, mouse antibody to Akt and horseradish peroxidase (HRP)‐conjugated anti‐rabbit and anti‐mouse antibodies were from Cell Signaling Technology (Danves, MA, USA).

### Preparation of human monocyte‐derived macrophages

Human citrated venous blood samples were obtained from the local blood transfusion centre (Lille, France). Following isolation of peripheral blood mononuclear cells by density centrifugation on Lymphoprep (Eurobio‐AbCys, Courtaboeuf, France), monocytes were purified with magnetic beads coupled to CD14, according to the instructions of the manufacturer (BD Biosciences, San Jose, CA, USA). Purity of the cell population was assessed by FACSCalibur flow cytometer (BD Biosciences) and found > 95%. Macrophages were then obtained by incubating freshly isolated monocytes (1 × 10^6^ cells·mL^−1^) for 5 days in RPMI 1640 medium (Lonza, Basel, Switzerland) supplemented with 10% (v/v) heat‐inactivated fetal calf serum and 10 ng·mL^−1^ of M‐CSF (Peprotech, Rocky Hill, NJ, USA). The experiments were undertaken with the understanding and written consent of each subject (EFS, NT/18/2015/092). The study methodologies conformed to the standards set by the Declaration of Helsinki and were approved by the local ethics committee (French Research Ministry, DC‐2008‐242).

### RNA isolation and real‐time RT‐PCR

Total RNA was isolated from 1 × 10^6^ cells using the NucleoSpin RNA II kit, according to the instructions of the manufacturer (Macherey‐Nagel, Düren, Germany). Reverse transcription was performed from 1 μg of total RNA using the Maxima First Strand cDNA Synthesis Kit for RT‐qPCR (Thermo Scientific). Synthetic primers for 3‐OSTs were described in Martinez *et al*. 28. Synthetic primers for HVEM, nectin‐2, Bcl‐2, Bcl‐XL and Bcl‐XS were designed using Primer‐Blast (NCBI) according to published sequences: HVEM (NM_003820.3), 5′‐GTGTGGTGTTTAGTGGATAC‐3′ (forward) and 5′‐ACAAATAGAAAACAGGAGCC‐3′ (reverse); nectin‐1 (NM_002855.4), 5′‐CATCGTCAACTACCACAT‐3′ (forward) and 5′‐CCTCAATGGTTACCTCAG‐3′ (reverse); nectin‐2 (NM_002856.2), 5′‐ ATGAGAGCTTCGAGGAAC‐3′ (forward) and 5′‐CGGAGATGGACACTTCAG‐3′ (reverse); Bcl‐2 (NM_000633.2), 5′‐GGATGCCTTTGTGGAACTGT‐3′ (forward) and 5′‐AGCCTGCAATTTTGTTTCAT‐3′ (reverse); Bcl‐XL (NM_138578.1), 5′‐TGAACAGGTAGTGAATGAAC‐3′ (forward) and 5′‐TCTCCTTGTCTACGCTTT‐3′ (reverse), and Bcl‐XS (NM_001191.2), 5′‐CAGAGCTTTGAACAGGATAC‐3′ (forward) and 5′‐GGTAGAGTGGATGGTCAG‐3′ (reverse; Eurogentec, Seraing, Belgium). Specificity of the primers was checked by semi‐quantitative RT‐PCR on a 2.5% (w/v) agarose gel. All of them amplified only one fragment of expected size, for which the sequence was confirmed (GATC Biotech, Constance, Germany). Real‐time PCR amplifications were performed using an Mx3000P Multiplex Quantitative PCR system (Agilent Technologies, Santa Clara, CA, USA). The transcript of hypoxanthine‐guanine phosphoribosyl transferase (HPRT) was used as a control to normalize the expression of the genes of interest. Each reaction of PCR consisted of 25 μL containing 2 μL of diluted cDNA sample (1 : 4), 12.5 μL of Maxima SYBR Green qPCR Master Mix (Thermo Scientific), 1 μL of forward primer (15 μm), 1 μL of reverse primer (15 μm) and 8.5 μL of water. It also included a nontemplate negative control to check for primer dimers. The conditions of PCR were as follows: 1 cycle of denaturation at 95 °C for 10 min, followed by 40 cycles of 30 s at 95 °C, 30 s at specific temperature of annealing (60 °C for all primer pairs) and 30 s at 72 °C. The fluorescence data were measured at the end of each cycle. A melting curve (55–95 °C at 1 °C interval) was constructed for each primer pair to check for the presence of one gene‐specific peak. The amplification efficiency of each primer pair was performed on serial dilutions of cDNA. Triplicate PCR reactions were prepared for each sample. The point at which the PCR product was first detected above a fixed threshold, termed cycle threshold (Ct), was determined for each sample, and the average Ct of triplicate samples was used for further analysis. The relative quantification of transcripts was calculated as described previously 50.

### Measurement of apoptosis

Following treatment of macrophages with recombinant HSV‐1 gD or CyPB (both at 1 μg·mL^−1^) for 8 h, apoptosis was induced by the addition of 0.5 μm staurosporin (Merck Millipore, Darmstadt, Germany) for 4 h. For apoptosis analysis, macrophages (2 × 10^5^ cells per point) were stained with annexin‐V and PI, using the Annexin‐V Apoptosis Detection Kit I (BD Biosciences) according to the manufacturer's instructions. Briefly, cells were harvested, rapidly washed with phosphate buffer (PBS: 20 mm Na phosphate, 150 mm NaCl, pH 7.2) and incubated in staining buffer in the presence of fluorescent annexin‐V and PI for 15 min at room temperature. Cell staining was visualized by flow cytometry using a FACSCalibur instrument and analysed with cellquest software (BD Biosciences), which allowed assignment of cells in viable (annexin‐V^−^/PI^−^), early apoptotic (annexin‐V^+^/PI^−^), late apoptotic (annexin‐V^+^/PI^+^) and necrotic (annexin‐V^−^/PI^+^) populations. Activation of caspase‐3 was also evaluated using the caspase‐3 assay fluorimetric kit (Sigma‐Aldrich), according to manufacturer's instructions. For each experimental condition, 2.5 × 10^5^ cells were lysed overnight at −80 °C and the enzymatic activity of caspase‐3 was measured thereafter by incubation of cell lysates with Ac‐DEVD‐AMC substrate. Specificity of the reaction was checked by the addition of Ac‐DEVD‐CHO caspase‐3 inhibitor. After 60 min of incubation at 37 °C, release of AMC product was monitored at 405 nm using a fluorimeter microplate reader. Values of absorbance were converted in caspase‐3 activity using an AMC standard curve and expressed in nanomoles of AMC released per min and per mL of cell lysate.

### Western immunoblotting

Human macrophages (1 × 10^6^ per point) were lysed in 75 μL lysis buffer (50 mm Tris‐HCl, 150 mm NaCl, 1% Triton X‐100, pH 8.0), supplemented with 1 mm Na orthovanadate, 10 mm sodium fluoride and protease inhibitor mixture (Roche Diagnostics, Meylan, France) for 3 h at 4 °C. The lysates were clarified by centrifugation at 10 000 ***g*** for 30 min at 4 °C, mixed with Laemmli buffer and boiled for 10 min. Proteins were separated by SDS/PAGE (10%, w/v) and transferred onto nitrocellulose membrane (Amersham, Uppsala, Sweden). The membrane was blocked for 1 h at room temperature in 20 mm Tris‐HCl, pH 7.6, 150 mm NaCl [Tris buffer saline (TBS)] supplemented with 0.05% (v/v) Tween‐20 and 5% (w/v) nonfat dry milk, and thereafter probed overnight with primary antibodies (1/3000) in TBS supplemented with 5% (w/v) nonfat dry milk. After washing, immunostaining was achieved using HRP‐conjugated secondary antibodies (1 : 10 000) and ECL detection (Amersham). Quantification of immunostaining intensity was performed using image j software. (National Institutes of Health, Bethesda, MD, USA, http://imagej.nih.gov/ij/, 1997–2016)

### RNA interference

Synthetic small‐interfering RNA (siRNA) duplexes with symmetric 3′‐deoxythymidine overhangs were used to carry out RNA interference. Macrophages were plated at a density of 1 × 10^6^ cells per well and transfected with siRNA (60 pmoles per well) using INTERFERin (Polyplus, Illkirch, France), according to the manufacturer's recommendations. The following siRNA sequences were designed (Sigma‐Aldrich) and tested for their efficiency to silence the expression of HVEM, nectin‐2, 3‐OST2 and CD147: si‐HVEM, 5′‐GCGAAGGUCUCACGAGGUCdTdT‐3′; si‐nectin‐2, 5′‐GUCACGGUCACCUGCAAAGdTdT‐3′; si‐3‐OST2, 5′‐CGGACAAGCACUUCUAUUUdTdT‐3′; si‐CD147, 5′‐GGUUCUUCGUGAGUUCCUCdTdT‐3′. A control siRNA duplex (MISSION^®^; Sigma) was used as negative control.

### Immunofluorescence staining

Following differentiation, macrophages were seeded on glass coverslips, washed once with PBS and fixed with 4% paraformaldehyde in 0.1 m sodium phosphate buffer (pH 7.2) for 30 min at room temperature. Coverslips were then washed three times with PBS, once with 1.5 m NaCl and then with PBS containing 0.1% saponin. Following blocking in PBS containing 0.5% BSA and 0.1% saponin, fixed cells were incubated in the presence of 10 μg·mL^−1^ of recombinant His‐tagged HSV‐1 gD (250 nm; Antibodies‐online, Aachen, Germany) in the same buffer, with or without CyPB. After 1 h of incubation at 4 °C, HSV‐1 gD binding was fluorescently detected by an Alexa 488‐conjugated anti‐His‐tag antibody (1 : 100; Qiagen, Hilden, Germany) in blocking buffer. Cells were also stained with 500 ng·mL^−1^ DAPI (Sigma‐Aldrich) for 10 min to visualize cell nuclei. Immunostaining was detected with an inverted Zeiss LSM 780 microscope (Oberkochen, Germany) with a 63× oil immersion lens at room temperature. Data were collected using the zeiss zen pro 2.1 software (Carl Zeiss Microscopy GmbH, Jena, Germany) and processed with image j software.

### Statistical analysis

Results are representative of at least three independent experiments conducted with human macrophages obtained from distinct donors. Statistical significance between the different values was analysed by Student's *t*‐test, with a threshold of *P* < 0.05 considered as significant.

## Author contributions

MD, FA and AD conceived and designed the experiments, MD, CH, FF, MC and AD performed the experiments, MD, FA and AD analysed the data and wrote the manuscript.
